# Source Control and Graft Preservation Using Negative Pressure Wound Therapy with Antibiotic Instillation: A Case Report

**DOI:** 10.7759/cureus.855

**Published:** 2016-10-31

**Authors:** Victoria Gilbert, Timothy Kelly, Robert Grossi

**Affiliations:** 1 General Surgery, Mount Sinai Beth Israel; 2 Vascular Surgery, Mount Sinai Beth Israel

**Keywords:** complex wounds, negative pressure wound therapy with instillation, bypass grafts

## Abstract

Negative pressure wound therapy (NPWT) is widely used to treat many types of complex wounds, and the advent of the instillation and dwell time (NPWTi-d) technique has enhanced this system with the addition of automated treatment with topical solutions. In the field of vascular surgery, NPWT is utilized to help close wounds over underlying grafts; however keeping these wounds free of infection and avoiding large reoperation when infection occurs remains a challenge. In this case report we present a patient who required acute intervention for limb ischemia, with a large wound created in the groin for anastomosis of a prosthetic graft bypass. Postoperatively, the wound became infected, and the challenge became balancing infection control and graft preservation with the patient’s multiple comorbidities including postoperative non-ST segment elevation myocardial infarction (NSTEMI). To avoid a large reoperation, we chose NPWTi-d with automated instillation of an antibiotic solution. There was no reinfection or return to the operating room (OR), the patient was discharged after four weeks and the wound closed on its own shortly thereafter. This case demonstrates that for high-risk surgical patients with known wound infections in the proximity of a bypass graft, NPWTi-d with antibiotic instillation may be an effective augmentation to current treatment strategies and may be considered as a stand-alone technique for wound closure in select cases.

## Introduction

Vascular grafts, particularly in the groin region, are vulnerable to infection and subsequent failure. This may result in life-threatening hemorrhage or sepsis and may require highly-morbid graft excision and revision. Studies have found that one to five percent of bypasses result in groin infections involving the graft, which is associated with amputation in 10–70% of cases and death in 10–20% of cases. Certain features are known to be associated with higher rates of reoperation and higher morbidity and mortality: a compromised anastomosis or incompetent graft, entire graft infection, known pseudomonal infection, and sepsis [1].

Several measures are employed in the postoperative period to help prevent infection and promote graft success and wound closure with demonstrated decrease in reinfection rates. Simple wet-to-moist dressings and frequent irrigations, interval wound debridement, muscle flap closure, and negative pressure wound therapy (NPWT) have all been utilized for this purpose [1-3]. Of these, NPWT has several advantages over the other methods. Continuously applied negative pressure delivers gentle mechanical debridement to underlying tissue, removes excess fluid, and stimulates cell proliferation and granulation [4]. NPWT dressings such as those used for a Vacuum-Assisted Closure system (V.A.C.®; KCI, Acelity, San Antonio, Texas) do not need to be changed nearly as often as wet-to-moist dressings, and NPWT has been shown to allow complete wound closure by secondary intention without the need for reoperation in many cases and to shorten time to closure when compared to simple dressings [2-3]. Additionally, avoiding operative muscle flap closure is ideal in patients who are not good reoperative candidates, such as those with poor nutritional status, high cardiac risk, and those with wounds with extensive fibrosis [1].

NPWT has been used for the past decade for vascular bypass graft preservation, with efficacy in patients with exposed and infected grafts [3]. In addition to continuous NPWT, certain augmentations have been implemented in efforts to optimize the therapy [4]. Of these, automated antibiotic instillation programmed into NPWT (NPWTi-d) combines the benefits of NPWT with those of irrigation and antisepsis. All three of these factors are crucial to wound healing, as they allow loosening of contaminants for proper perfusion, granulation tissue formation, and eventual wound closure more effectively than NPWT alone [5]. In this case report we describe our use of NPWT with instillation of antibiotic solution to promote both infection control and graft preservation as an effective means of uncomplicated wound healing by secondary intention over a vascular graft. Informed consent was obatined from the patient for this study.

## Case presentation

An 86-year-old male with a history of hypothyroidism, hypertension, hyperlipidemia, congestive heart failure, coronary artery disease with prior MI, atrial fibrillation on systemic anticoagulation, and peripheral arterial disease requiring multiple past interventions including a right below knee amputation presented to the outpatient vascular clinic with left foot pain associated with new ulcers on his left lower extremity. On exam he was found to have left lower extremity ischemia in the distribution of the dorsalis pedis artery without neuromuscular compromise. He was sent immediately to the hospital and started on intravenous (IV) heparin and IV antibiotics and taken to the OR on hospital day two, where a left lower extremity angiogram showed distal superficial femoral artery occlusion requiring common femoral-to-popliteal bypass with a synthetic graft (GORE® PROPATEN®, W.L. Gore, Flagstaff, Arizona). His groin and leg wounds were closed primarily at the end of the case. 

**Figure 1:**
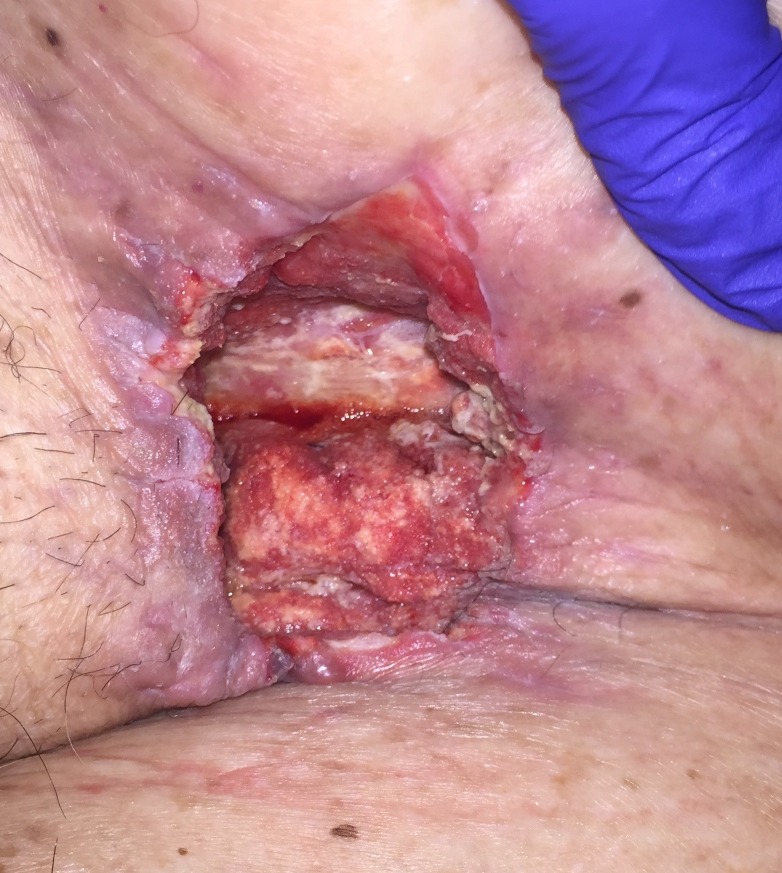
POD 18

**Figure 2:**
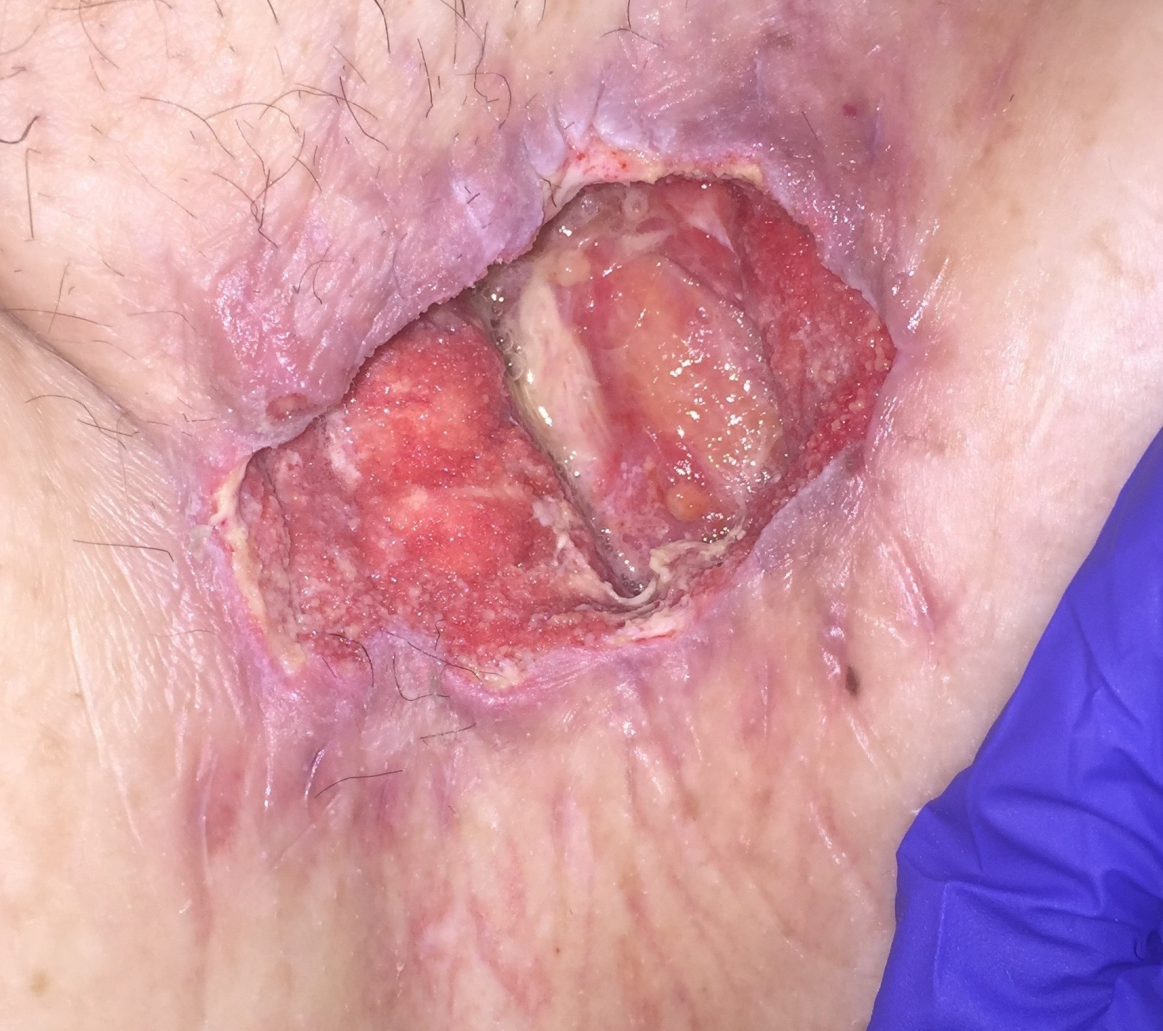
POD 22

**Figure 3:**
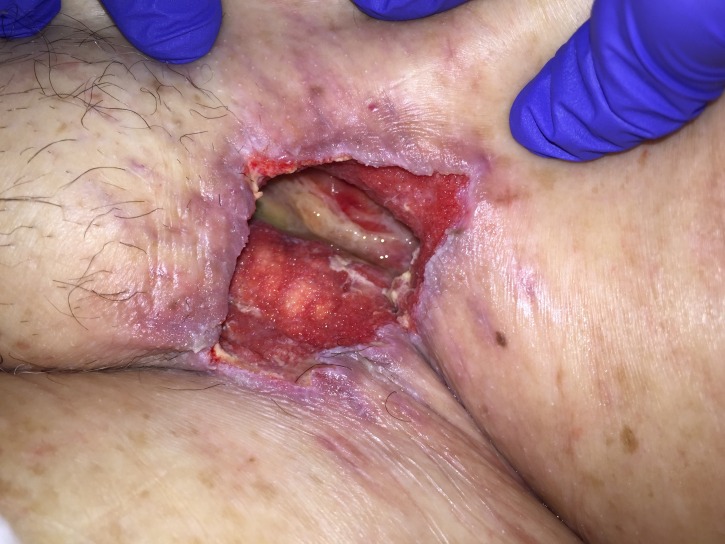
POD 25

His immediate postoperative course was complicated by NSTEMI on postoperative day one (POD 1), which was managed medically. On POD 11 he was noted to have pus draining from his left groin wound along with a persistent leukocytosis and methicillin-resistant Staphylococcus aureus (MRSA) bacteremia. He was taken to the OR for wound debridement and washout and found to have exposed graft and pus in the wound. On POD 13 he returned to the OR for further washout, this time with VAC placement (V.A.C.®, KCI, Acelity, San Antonio, Texas) with antibiotic instillation. The NPWTi-d system involved placing a non-adherent dressing (ADAPTIC™, Acelity, San Antonio, Texas) followed by a reticulated open-cell foam (V.A.C. VERAFLO™ Dressing, Acelity, San Antonio, Texas) covered by a semi-occlusive dressing (V.A.C.® Advanced Drape, Acelity, San Antonio, Texas) connected to the negative pressure device using a combined suction and solution delivery tubing (VERAT.R.A.C.™ Pad and Tubing Set, Acelity, San Antonio, Texas). VAC therapy was set at 125 mmHg, interrupted every two hours by 10 minutes of irrigation and dwell time with a solution of bacitracin/polymyxin in normal saline. The volume of instillation solution administered was calculated in the following manner: largest wound diameter in cm x wound depth in cm = cc of instillation fluid. VAC changes were performed every two days for the first two weeks postoperatively and every two to three days thereafter until discharge. Each dressing change involved gentle irrigation with normal saline followed by hydrogen peroxide to debride areas with fibrinous exudate before a new non-adherent dressing and VAC dressings were placed. At all dressing changes increased granulation tissue along with the absence signs of infection were noted, as can be appreciated in Figures 1-3.

**Figure 4:**
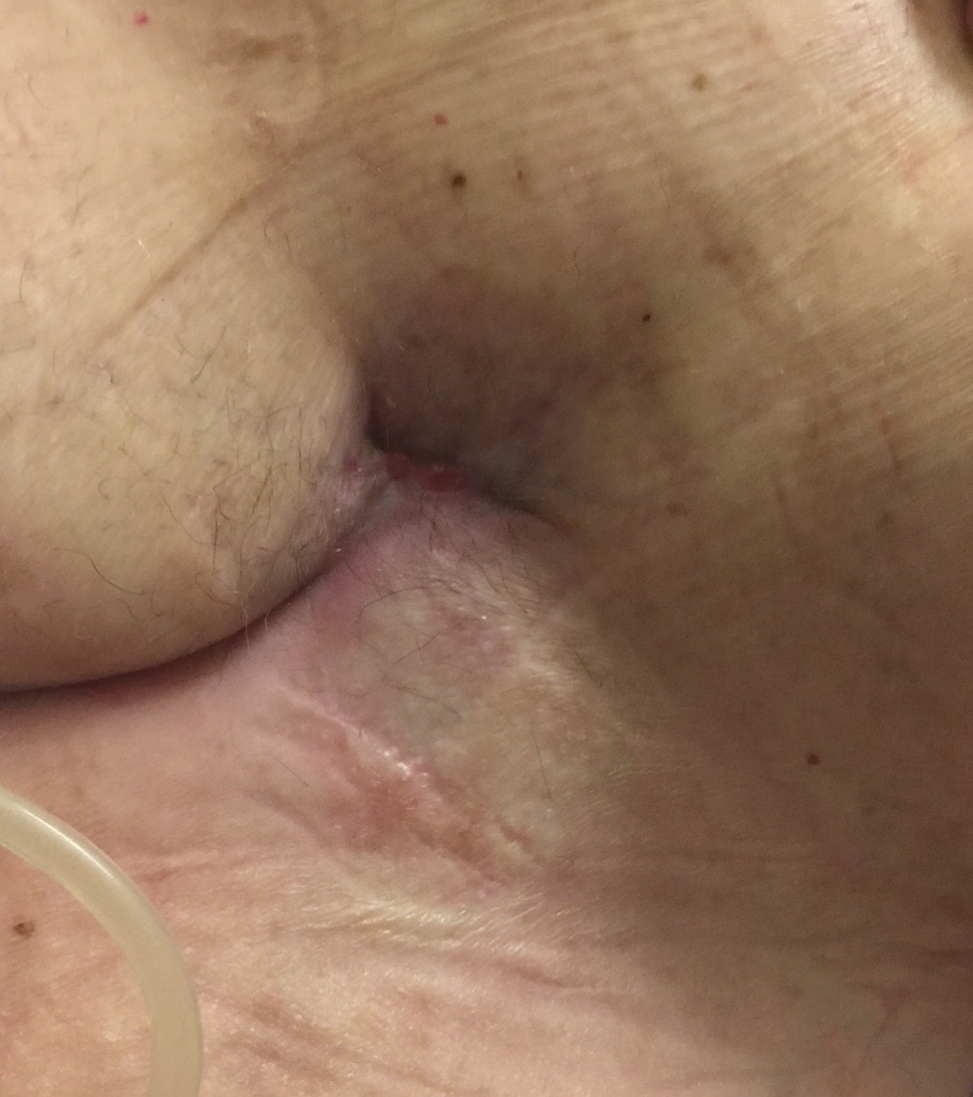
14 Weeks Postoperatively

A peripherally inserted central catheter (PICC) line was placed for a six-week course of IV antibiotics. The patient was discharged on POD 28 at which point the VAC was converted to a NPWT VAC without instillation. On POD 40 the patient presented to the outpatient office without complaints, and the wound VAC was removed and the wound was deemed shallow enough to switch to simple dressings. In a telephone follow-up at 14 weeks the patient noted that his wound was entirely closed and he was asymptomatic, without infection, and able to continue rehabilitation. Figure 4 shows appearance of the closed groin wound at this follow-up. His graft has remained patent throughout this entire course.

## Discussion

Vascular grafts in the groin region are subject to high rates of infection and failure. Groin wounds are especially susceptible to infection in patients with diabetes, obesity, immunocompromised state, and reoperation, and pose a great risk to the competence of underlying grafts if the groin infections are deep [2]. Certain clinical features are known to be associated with higher rates of reoperation and graft excision and higher morbidity and mortality: a compromised anastomosis or incompetent graft, entire graft infection, known pseudomonal infection, and sepsis [2]. Reoperation has long been considered the management for graft infection—graft excision and revision/reconstruction and/or closure with a myocutaneous flap. Commonly, a conflict arises between the morbidity of these operations and the need for definitive treatment, as most patients with graft infections have significant comorbidities or are critically ill at the time of infection [2].

Due to the high incidence of graft infection in patients with contraindications to reoperation, conservative graft preservation has become a priority in postoperative wound care. NPWT has for many years provided an effective alternative for graft preservation in select cases, with lower reinfection rates than local debridement and delayed muscle flap closure [1-2]. NPWT is known to decrease the time to granulation tissue formation which is a crucial component to avoiding infection or graft compromise [2]. In addition, muscle flap failure rates of up to 35% have been documented, and many of the patients in need of definitive wound care and closure are not ideal candidates for a muscle flap surgery, such as the critically ill and those with high cardiac risk [1].

In 2012 KCI debuted the V.A.C. VERAFLO™ system as an augmentation to Vacuum-Assisted-Closure (V.A.C.®) NPWT technology and as a more integrated adaptation of its earlier VAC instillation capabilities [4]. NPWT with irrigation and dwell time (NPWTi-d) was described previously with promising results as it was first studied extensively in orthopedic and diabetic wounds [3]. In addition to NPWT’s benefits of removal of excess fluid, promotion of perfusion, and increased granulation tissue, instillation therapy is thought to lower wound fluid viscosity, facilitating the washout of necrotic tissue, fibrin, and clots. While simple NPWT confers some degree of mechanical stress to stimulate underlying cell proliferation, NPWTi-d applies it more gently and allows new cells to be laid down on healthy tissue [3, 6]. Cycling between negative pressure and instillation stimulates frequent debridement which has been shown to remove inhibitory factors such as metalloproteases and break up the glycocalyx that causes biofilms to form, which are much more resistant to antisepsis [3]. In a study of 131 cases of wounds treated with NPWTi-d, investigators observed increased granulation tissue production, more rapid granulation, and successful eventual closure in 98% of cases whereas this process had stalled or failed conventional NPWT. No vascular grafts were included in this study; however, it was noted that exposed orthopedic hardware and bone were more rapidly and more completely covered compared to conventional NPWT [4].

As illustrated in this case report, NPWTi-d automates the cycles between negative pressure therapy and volumetric irrigation with topical solution with dwell time, reducing the overall number of dressing changes and the need for OR debridement [4]. This therapy is minimally invasive and highly convenient in ill patients and those immobile or too at risk to return to the operating room [7]. In a study of 142 patients with poorly healing wounds, 74 treated with traditional NPWT and 68 with NPWTi-d, statistically significant decreases were noted in number of returns to OR, length of hospital stays and time to definitive closure, with significant increases in percentage of wounds closed as well as bacterial culture improvement [5]. Although the majority of data regarding NPWTi-d is gained from orthopedic and extremity wounds, promising outcomes have been noted in case reports presenting the use of NPWTi-d in wounds associated with pleural empyema and mediastinitis, both types similar to wounds over vascular grafts in their delicate underlying structures and similar high risk patient populations [6-7]. Furthermore, NPWTi-d has been shown to reduce the time to wound closure, shorten the overall hospital stay, and thereby decrease overall costs [8].

Further studies, particularly randomized control trials, are needed to examine the impact of NPWTi-d on protecting underlying vascular grafts. These studies should also examine the many variables that are encountered when managing these types of wounds. Most cases utilize some type of non-adherent dressing between the graft and VAC sponge to reduce the risk of irritation to the exposed graft; further evaluation may be needed to compare types of non-adherent dressings versus a “double-sponge” system (i.e. with V.A.C. WHITEFOAM™ Dressing) [2]. Saline or chemical irrigation as well as mechanical bedside debridement at the time of dressing change is often done as a means of preparing the wound bed for the subsequent VAC placement. In our case, hydrogen peroxide was used as an adjunct for loosening debris in areas of the wound which were well-granulated with overlying fibrinous exudate; however, this was an empirical treatment and further studies are needed to evaluate the role, benefits, and risk of this and other adjunctive debridement methods with the NPWTi-d system. Additionally, much attention has been paid to the concept of the biofilm and the effectiveness of NPWTi-d in helping to prevent its formation [4]. However, the type of topical instillation solution has not been widely studied. Interestingly, in one study comparing normal saline vs antiseptic solution instillation, there was no statistical significance in wound healing between the two groups, although they were both statistically superior to simple NPWT [9].

## Conclusions

Negative pressure wound therapy with antibiotic instillation (NPWTi-d) appears to be a safe and effective method for infection prevention and control as well as vascular graft preservation in the groin region, as illustrated by our case report. Additionally, this report describes the successful use of this method as a stand-alone treatment for both wound therapy and closure.

## References

[1] Dosluoglu HH, Schimpf DK, Schultz R, Cherr GS (2005). Preservation of infected and exposed vascular grafts using vacuum assisted closure without muscle flap coverage. J Vasc Surg.

[2] Berger P, de Bie D, Moll FL, Borst GJ (2012). Negative pressure wound therapy on exposed prosthetic vascular grafts in the groin. J Vasc Surg.

[3] Gabriel A, Shores J, Heinrich C, Baqai W, Kalina S, Sogioka N, Gupta S (2008). Negative pressure wound therapy with instillation: a pilot study describing a new method for treating infected wounds. Int Wound J.

[4] Brinkert D, Ali M, Naud M, Maire N, Trial C, Teot L (2013). Negative pressure wound therapy with saline instillation: 131 patient case series. Int Wound J.

[5] Kim PJ, Attinger CE, Steinberg JS, Evans KK, Powers KA, Hung RW, Smith JR, Rocha ZM, Lavery L (2014). The impact of negative-pressure wound therapy with instillation compared with standard negative-pressure wound therapy: a retrospective, historical, cohort controlled study. Plast Reconstr Surg.

[6] Karaca S and Kalangos A (2015). Vacuum-assisted closure (VAC)-Instill with continuous irrigation for the treatment of Mycoplasma hominis mediastinitis. Int Wound J.

[7] Hofmann HS, Neu R, Potzger T, Schemm R, Grosser C, Szoke T, Sziklavar Z (2015). Minimally invasive vacuum-assisted closure therapy with instillation (Mini-VAC-Instill) for pleural empyema. Surg Innov.

[8] Gabriel A, Kahn K, Karmy-Jones R (2014). Use of negative pressure wound therapy with automated, volumetric instillation for the treatment of extremity and trunk wounds: clinical outcomes and potential cost-effectiveness. Eplasty.

[9] Kim PJ, Attinger CE, Oliver N, Garwood C, Evans KK, Steinberg JS, Lavery LA (2015). Comparison of outcomes for normal saline and an antiseptic solution for negative-pressure wound therapy with instillation. Plast Reconstr Surg.

